# Safety, Tolerability, and Efficacy of Tocilizumab in Rheumatoid Arthritis: An Open-Label Phase 4 Study in Patients from the Middle East

**DOI:** 10.1155/2015/975028

**Published:** 2015-05-19

**Authors:** Mohammed Hammoudeh, Adel Al Awadhi, Eman Haji Hasan, Maassoumeh Akhlaghi, Arman Ahmadzadeh, Bahar Sadeghi Abdollahi

**Affiliations:** ^1^Weill Cornell Medical College in Qatar and Hamad General Hospital, Doha, Qatar; ^2^Department of Medicine, Faculty of Medicine, Kuwait University, Kuwait City, Kuwait; ^3^Rheumatic Disease Unit, Alamiri Hospital, Kuwait City, Kuwait; ^4^Rheumatology Research Center, Shariati Hospital, Tehran University of Medical Sciences, Tehran, Iran; ^5^Rheumatology Ward, Loghman Hakim University Hospital, Shahid Beheshti University of Medical Sciences, Tehran 14155 6153, Iran

## Abstract

This open-label study investigated the safety and efficacy of tocilizumab in Middle Eastern patients with rheumatoid arthritis (RA). Patients whose Disease Activity Score based on 28 joints (DAS28) was >3.2 received tocilizumab 8 mg/kg intravenously every 4 weeks for 24 weeks. Patients receiving aTNF ± nonbiologic disease-modifying antirheumatic drug(s) (DMARD(s)) switched to tocilizumab; patients receiving nonbiologic DMARD monotherapy added tocilizumab. Primary end points were adverse events (AEs), serious AEs (SAEs), and laboratory parameters; secondary end points were DAS28, Health Assessment Questionnaire-Disability Index, C-reactive protein (CRP), and erythrocyte sedimentation rate (ESR). Eighty-eight of 95 patients completed 24 weeks. Overall, 125 AEs were reported in 43 (45%) patients; the most common were increased hepatic enzymes (16%) and cholesterol (11%). Eight patients experienced SAEs. Significant changes from baseline to week 24 occurred for hemoglobin, neutrophils, platelets, total cholesterol, and liver enzymes (*P* < 0.05). DAS28, CRP, and ESR decreased significantly from baseline at each visit (*P* < 0.0001). At week 24, the proportions of patients reporting DAS28 clinically meaningful improvement (decrease ≥1.2), low disease activity (DAS28 ≥2.6 to ≤3.2), and remission (DAS28 <2.6) were 92%, 23%, and 64%, respectively. Safety and efficacy of tocilizumab were consistent with values reported in Western patients.

## 1. Introduction

Rheumatoid arthritis (RA) affects approximately 0.5% to 1.0% of the population in Western countries [1]. Information regarding its prevalence in the Middle East is sparse, but recent estimates ranging from 0.2% to 1.0% have been reported in patients from different regions of Iran [2]. Early evidence [3, 4] in Middle Eastern patients with RA suggests they may have milder disease than Western patients; low incidences of rheumatoid factor (RF) positivity (60%) and rheumatoid nodules (7%) are reported in Arab patients [3], and RF positivity of 66% is reported in patients from Iran [4]. More recent evidence suggests that disease severity in patients from the Middle East is comparable to that in RA patient cohorts from the United States and Europe, with similar RF positivity of approximately 75% reported [5–7]. A clinical remission rate of 58% has been reported by a retrospective study in patients from Iran treated with disease-modifying antirheumatic drugs (DMARDs) over 5 years; however, the criteria used for remission were not described [4]. A recent study [8] in patients with RA from Qatar shows a 49% remission rate according to the Disease Activity Score based on 28 joints (DAS28 <2.6) and a 15% low disease activity rate (DAS28, 2.6–3.2). These rates are higher than those reported in other Middle Eastern countries and are possibly related to greater use of biologics [8].

Middle Eastern and Western patients with RA have genetic differences that may influence disease activity and severity. For example, the profile of HLA-DR antigens is different between these groups. Middle Eastern patients predominantly have HLA-A10, B8, B21, DR3, and DR1 antigens rather than HLA-DR4, which is associated with RA in the West [9, 10]. Differences in genetics could potentially influence treatment outcomes and adverse events in patients with RA [11]. Therefore, it is possible that tocilizumab treatment could have different effects in different HLA configurations, with a potential for varying results between Middle Eastern and Western patients.

Interleukin-6 (IL-6) is a multifunctional, proinflammatory cytokine implicated in the pathogenesis of RA [12, 13]. Tocilizumab, a humanized monoclonal antibody, binds to membrane-bound and soluble IL-6 receptors and inhibits IL-6 signaling pathways [14, 15]. Tocilizumab is approved in the United States and Europe as monotherapy or as combination therapy with methotrexate for the treatment of adults with moderate to severe RA who are intolerant of or resistant to DMARDs or antitumor necrosis factor (aTNF) agents [16, 17]. Phase 2 and 3 studies demonstrated that tocilizumab 8 mg/kg every 4 weeks resulted in the highest efficacy response rate with an acceptable safety profile [18–23].

The current study was conducted across Bahrain, Iran, Kuwait, Qatar, and UAE, representing a region of the world for which the efficacy and safety of tocilizumab have not been specifically investigated.

## 2. Methods

### 2.1. Study Design

The Safety, Tolerability, and Efficacy of Actemra (Tocilizumab) in Rheumatoid Arthritis (STEARA) study (ClinicalTrials.gov NCT01089023) was an open-label, single-arm, phase 4 study conducted at 7 sites throughout 5 countries (Bahrain, Iran, Kuwait, Qatar, and UAE) between January 13, 2010, and June 20, 2011. The primary objective of the study was to assess the safety and tolerability of tocilizumab monotherapy or combination therapy with nonbiologic DMARDs in patients with moderate to severe active RA. Secondary objectives were to assess the efficacy of tocilizumab monotherapy or combination therapy with nonbiologic DMARDs.

Tocilizumab was administered intravenously (60-minute infusion) at a dose of 8 mg/kg every 4 weeks on an outpatient basis. Patients received 6 infusions over a treatment duration of 24 weeks. Each treatment regimen depended on the patient's background DMARD therapy at baseline. For the patient receiving a nonbiologic DMARD plus aTNF therapy at baseline, aTNF therapy was discontinued and tocilizumab was initiated without a washout period, consistent with routine clinical practice. For the patient receiving a nonbiologic DMARD as monotherapy, tocilizumab was added to the existing regimen. For the patient receiving aTNF monotherapy, aTNF therapy was discontinued. Tocilizumab was initiated after a waiting period based on local guidelines or, if there were none, at 2 to 5 times the half-life of the specific aTNF agent, in accordance with international practices.

The study was conducted in accordance with the principles of good clinical practice and was approved by the institutional review board at each of the 7 study sites. To be included in the study, patients had to be able and willing to give written informed consent. Patient consent was obtained to allow data to be included in the study in an anonymous manner and with full respect for patient confidentiality.

### 2.2. Study Population

Adult patients (18 years of age or older) with RA diagnosed ≥6 months earlier and with moderate to severe disease activity based on 28 joints (DAS28 >3.2) [24] were included. Patients who had a known history of or who had active tuberculosis, hepatitis B, or hepatitis C or who were positive for hepatitis B surface antigen (HBsAg) or hepatitis C antibody were excluded from the study. In each study center, patients were screened for latent tuberculosis before biologics were used, in accordance with local guidelines or good clinical practice. Patients with latent tuberculosis were treated with standard antimycobacterial therapy for at least 4 weeks before tocilizumab was initiated, and they had to have negative chest X-ray findings at screening. Patients had to be receiving at least 1 nonbiologic DMARD and/or aTNF therapy at a stable dose for 8 weeks or longer before the baseline visit. Stable doses of oral corticosteroids (≤10 mg/day prednisone or equivalent) and nonsteroidal anti-inflammatory drugs were allowed. Patients could not have previously received tocilizumab.

### 2.3. Assessments

The primary study end point was the safety and tolerability of tocilizumab. Safety was assessed as the incidence of adverse events (AEs), including serious AEs (SAEs), during 24 weeks of tocilizumab monotherapy or combination therapy with continued nonbiologic DMARD therapy. The numbers and percentages of patients who discontinued treatment because of AEs and SAEs were assessed. Transaminase elevations were assessed as proportions of patients with levels of alanine aminotransferase (ALT) and aspartate aminotransferase (AST) ≥3x the upper limit of normal (ULN). Lipid elevations were assessed as proportions of patients with high-density lipoprotein (HDL), low-density lipoprotein (LDL), total cholesterol, and triglyceride elevations according to Adult Treatment Panel (ATP) III Guidelines. Hematology (neutrophil counts, percentages of lymphocytes, platelet counts, white blood cell counts, and hemoglobin) and blood chemistry (albumin, alkaline phosphatase, indirect and total bilirubin, and total protein levels) parameters were assessed. Incidences of major adverse cardiac event (MACE) and stroke were also investigated during the study.

Efficacy assessments were secondary end points and included the numbers and percentages of patients achieving clinically meaningful improvement in DAS28 (reduction of ≥1.2 units), low disease activity (DAS28 ≥2.6 to ≤3.2), and remission (DAS28 <2.6). Time to clinically meaningful improvement, low disease activity, or remission was also assessed. Erythrocyte sedimentation rate (ESR) and C-reactive protein (CRP) levels were assessed at each visit. Physical function was assessed using the Heath Assessment Questionnaire-Disability Index (HAQ-DI) [25]. Clinically meaningful improvement in HAQ-DI was defined as a decrease of at least 0.22 units from baseline. HAQ-DI <1 was considered mild disability, HAQ-DI between 1 and 2 was considered moderate disability, and HAQ-DI ≥2 was considered severe disability.

### 2.4. Statistical Analyses

The analysis population included all patients who were administered study drug. The Friedman test (nonparametric repeated-measures analysis of variance [ANOVA]) or the Mann-Whitney *U* test or both were performed for determination of AE incidence and assessment of DAS28 and HAQ-DI. Change from baseline values for CRP and ESR levels was assessed using ANOVA.

## 3. Results

### 3.1. Patient Disposition

**Figure 1:**
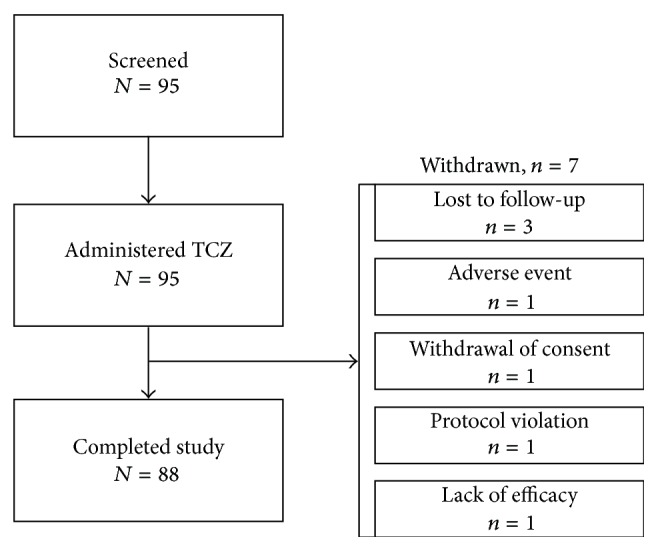
Patient disposition. TCZ, tocilizumab.

All 95 patients screened for the study were administered study medication; 93 patients received tocilizumab in combination with ≥1 DMARD, and 2 received tocilizumab as monotherapy. Of these patients, 88 completed the study to week 24 and 7 discontinued before study end. Reasons for withdrawal were AE (*n* = 1), withdrawal of consent after 2 infusions of tocilizumab (*n* = 1), protocol violation (*n* = 1), being lost to follow-up (*n* = 3), and insufficient therapeutic response (*n* = 1) (Figure 1). The mean age of patients was 44.9 years. Eighty-two percent of patients were female. Most patients (73%) were white, 3% were black, and the remaining 24% were of other races. Mean DAS28 score at baseline was 6.1 (Table 1).

### 3.2. Safety

AEs were reported by 43 (45.3%) patients who experienced a total of 125 events (Table 2). The most commonly occurring AEs were reported under the system organ class (SOC) of investigations, infections and infestations, and metabolism and nutrition disorders. The most commonly reported AEs under the SOC of investigations were increased hepatic enzymes in 15 patients (15.8%) and increased blood cholesterol level in 10 patients (10.5%). Most patients reporting AEs had events of mild or moderate intensity; 5 patients (5.3%) reported severe AEs.

AEs deemed related to study medication were reported in 36.8% of patients (*n* = 35). The most common of these were investigations (22.1%), metabolism and nutrition disorders (7.4%), and infections (5.3%) (Table 2). No significant difference was observed in the incidences of AEs by visit (*P* = 0.1762).

Eleven SAEs were experienced by 8 patients (8.4%) in the study. Nine SAEs were considered possibly related to study treatment (Table 2) and included hypercholesterolemia, elevated liver enzymes, and hypertriglyceridemia. One patient experienced massive extension of a mandibular infection to cervicofacial soft tissue that was considered possibly treatment related; the patient recovered with therapy. Another patient experienced a relapse of RA, also considered possibly treatment related, which led to persistent disability.

One patient experienced an SAE of acute renal impairment; the patient had a history of diabetes and hypertension, and the primary investigator deemed the event not related to study medication. The patient withdrew from the study after 4 weeks and continued to experience renal impairment. This was the only AE that led to withdrawal from the study. No AEs leading to dose modification, no MACE, and no deaths occurred during the study.

Significant changes from baseline to week 24 in laboratory parameters were observed for hematology assessments, including significant effects on hemoglobin, lymphocyte percentage, neutrophil count, white blood cell count, and platelet count (*P* ≤ 0.001). There were also significant effects on blood chemistry measurements, including albumin, alkaline phosphatase, indirect bilirubin, total bilirubin, and total protein levels (*P* ≤ 0.01; Table 3). Mean changes from baseline to week 24 in lipid profiles were not significant except for total cholesterol, with a mean (standard deviation (SD)) change of 0.24 (0.86) mmol/L (*P* = 0.016). There were no significant lipid elevations according to ATP III criteria for HDL, LDL, total cholesterol, or triglycerides from baseline to any of the subsequent visits (all *P* > 0.05). Significant elevations in liver enzymes were observed, with mean ALT and AST levels increasing from baseline to week 24 by 11.5 (SD 17.0; *P* < 0.0001) and 4.9  (SD 11.4; *P* = 0.011) IU/L, respectively. One patient had an ALT/AST elevation ≥3x ULN at week 4, with levels returning to <3x ULN by the following study visit. No dose modification or interruption was reported, and ALT levels returned to <3x ULN by the following study visit. This patient completed the study.

### 3.3. Efficacy

**Figure 2:**
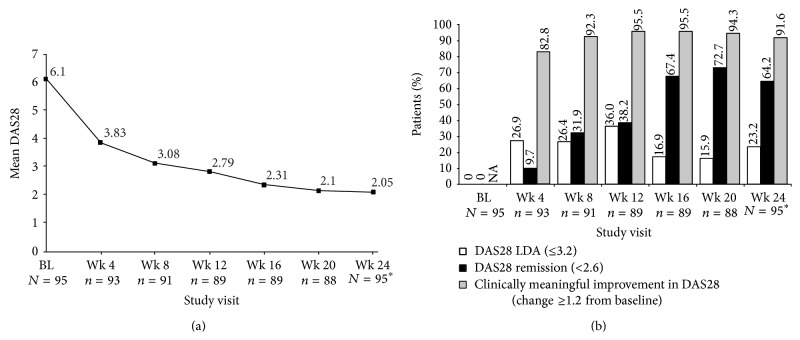
DAS28 to week 24. (a) Mean score over time. (b) Proportions of patients achieving DAS28 low disease activity (LDA; ≤3.2), DAS28 remission (<2.6), and clinically meaningful changes from baseline over time (change ≥1.2 from baseline). NA, not applicable. (a) *P* < 0.0001 for all visits after baseline versus baseline, based on Wilcoxon signed rank test. *n* = number of patients with treatment administered at that visit. ^*∗*^Follow-up visit for all patients. (b) *n* = number of patients with treatment administered at that visit. ^*∗*^Follow-up visit for all patients.

The mean DAS28 score decreased from 6.1 at baseline to 2.05 at week 24 (mean change from baseline, –4.06; Figure 2(a)). Decreases from baseline were statistically significant at all postbaseline visits (*P* < 0.0001; Figure 2(a)). Similar reductions over time were observed for tender joint count (TJC) and swollen joint count (SJC). Mean TJC decreased from 13.28 at baseline to 1.19 (change of –12.1; *P* < 0.0001 versus baseline), and mean SJC decreased from 8.6 at baseline to 1.12 (change of –7.46; *P* < 0.0001 versus baseline) at 24 weeks. Clinically meaningful improvement in DAS28 (reduction ≥1.2 units from baseline) was achieved in 92% of patients at week 24, with a mean time to response of 36.4 ± 2.4 days (Figure 2(b)). High disease activity observed in 63 (66.3%) patients at baseline decreased over time, and no patients demonstrated high disease activity at the end of the study. In total, 22 (23.2%) patients had low disease activity (DAS28 ≥2.6 to ≤3.2) at week 24. The highest proportion of patients reporting low disease activity occurred at week 12 (36%). The proportion of patients with disease remission (DAS28 <2.6) increased with treatment visit, peaking at week 20 (73%) (Figure 2(b)). Overall, 61 (64.2%) patients achieved disease remission at 24 weeks. Mean time to DAS28 low disease activity and remission was 75 and 90 days, respectively.

**Figure 3:**
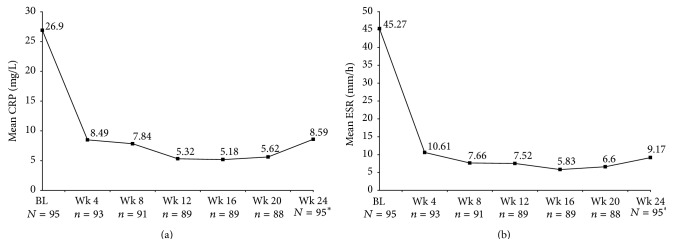
CRP (a) and ESR (b) to week 24. CRP, C-reactive protein; ESR, erythrocyte sedimentation rate. (a) *P* < 0.001 for all visits after baseline versus baseline, based on least square pairwise comparison. *n* = number of patients with treatment administered at that visit. ^*∗*^Follow-up visit for all patients. (b) *P* < 0.001 for all visits after baseline versus baseline, based on least square pairwise comparison. *n* = number of patients with treatment administered at that visit. ^*∗*^Follow-up visit for all patients.

The numbers of patients with abnormal values for ESR and CRP at baseline were 80 (84%) and 57 (60%), respectively. By week 4, only 9 (10%) patients had abnormal ESR values and only 10 (11%) patients had abnormal CRP values; these were sustained through week 24 (12% and 11% of patients had abnormal ESR and CRP values, resp., at study end). Mean ESR and CRP levels decreased significantly after the baseline visit, with the lowest levels observed at week 16 for both (*P* < 0.0001 versus baseline). Mean (SD) CRP decreased from 26.9 (34.4) mg/L at baseline to 8.6 (21.7) mg/L, and mean (SD) ESR decreased from 45.3 (29.2) mm/h at baseline to 9.17 (12.3) mm/h at 24 weeks (Figures 3(a) and 3(b)). These changes from baseline were statistically significant at every visit from week 4 to week 24 (*P* < 0.0001; Figures 3(a) and 3(b)).

A clinically meaningful HAQ-DI response (decrease of ≥0.22 units from baseline) was achieved by 89.5% of patients. The proportion of patients in the severe disability category (HAQ-DI ≥2) was the highest at baseline (24%) and decreased to 3% at 24 weeks. The proportion of patients in the mild disability category (HAQ-DI <1) increased from 14% at baseline to 72% at the end of the treatment period (week 24).

## 4. Discussion 

The overall safety, tolerability, and efficacy profile of tocilizumab in this study was comparable to that reported from studies conducted in the United States and Europe [19–22]. The current study is the first to investigate efficacy responses and side effects of RA treatment with tocilizumab exclusively in patients from Middle Eastern countries. The study population and treatment paradigm are reflective of routine clinical practice in that inclusion and exclusion criteria were relaxed compared with earlier randomized controlled trials, and no washout period was required for switching from aTNF therapy to tocilizumab.

The percentage of AEs reported in the current study was slightly lower than that reported in previous studies, but the percentage of SAEs was similar. In the tocilizumab pivotal phase 3 trial program, the reported overall rates of AEs and SAEs ranged from 69% to 87% and from 4.1% to 7.4% [19–22], respectively, compared with 45.3% and 8.4%, respectively, in the current study. Studies with a similar design, which enrolled patients representative of routine clinical practice, reported rates of 77% and 8%, respectively, in ACT-SURE [26] and 84.6% and 5.2% (SAEs related to study drug), respectively, in TAMARA [27]. Most AEs were reported in the SOC investigations; 15.8% of patients experienced elevation of liver enzymes, though investigators judged elevations to be related to treatment with tocilizumab in only 9.5% of patients. One patient withdrew from treatment because of acute renal impairment; this patient had a history of diabetes and hypertension. General physical abnormalities were recorded at the baseline visit, and the event was not considered related to study medication.

Response rates to tocilizumab 8 mg/kg in the current study followed patterns similar to those in other studies conducted in Western patients with RA. Improvements were demonstrated in DAS28, CRP, ESR, and HAQ-DI [19–23]. However, these randomized controlled trials reported DAS28 <2.6 in 30% to 47% of patients with inadequate responses to DMARD [19, 21, 23] and in 30% of patients with inadequate responses to tumor necrosis factor receiving tocilizumab 8 mg/kg [20] compared with 64% in the current open-label study. Other studies with a design similar to that of the current study reported comparable response rates; DAS28 <2.6 was reported in 62% of patients treated with tocilizumab in the ACT-SURE study [26] and in 53% of patients in the TAMARA study [27]. Clinically meaningful improvement in DAS28 was achieved by 92% of patients in the current study and 79% of patients in TAMARA [27].

In conclusion, the current study demonstrates that the efficacy and safety of tocilizumab in patients with RA from the Middle East are comparable to those previously demonstrated in similar studies conducted in Western patients with RA.

## Figures and Tables

**Table 1:** Demographics and baseline disease characteristics.

	Tocilizumab
	*N* = 95
Patient characteristics	
Sex, *n* (%)	
Female	78 (82.1)
Male	17 (17.9)
Race, *n* (%)	
White	69 (72.6)
Black	3 (3.2)
Other	23 (24.2)
Smokers, *n* (%)	4 (4.2)
Age, years, mean (SD)	44.9 (13.7)
BMI, kg/m^2^, mean (SD)	28.1 (5.4)
Disease characteristics	
DAS28, mean (median [range])	6.1 (5.8 [3.4, 8.8])
Tender joint count, mean	13.28
Swollen joint count, mean	8.6
HAQ-DI, mean (SD)	1.6 (0.6)
CRP, mg/L, mean (SD)	26.9 (34.4)
ESR, mm/h, mean (SD)	45.3 (29.2)
Concomitant medication used by >10%, *n* (%)	
Immunosuppressants	86 (90.5)
Methotrexate	83 (87.4)
Prednisolone	47 (49.5)
Methylprednisolone	6 (6.3)
Prednisone	7 (7.4)
aTNF agent (etanercept or adalimumab)	5 (5.3)
Antianemia preparations	65 (68.4)
Anti-inflammatory and antirheumatic products	50 (52.6)
Vasoprotectives	46 (48.4)
Antiprotozoals	37 (38.9)
Mineral supplements	35 (36.8)
Vitamins	30 (31.6)
Antidiarrheals, intestinal anti-inflammatory/anti-infective agents	29 (30.5)
Drugs for acid-related disorders	27 (28.4)
Lipid-modifying agents	21 (22.1)
Drugs for treatment of bone diseases	14 (14.7)
Agents acting on the renin-angiotensin system	12 (12.6)
Drugs used in diabetes	11 (11.6)

BMI, body mass index; CRP, C-reactive protein; DAS28, Disease Activity Score based on 28 joints; ESR, erythrocyte sedimentation rate; HAQ-DI, Health Assessment Questionnaire-Disability Index; SD, standard deviation.

**Table 2:** Adverse events by system organ class.

	Tocilizumab
	*N* = 95
Patients with AEs	43 (45.3)
Patients with treatment-related AEs	35 (36.8)
Investigations	21 (22.1)
Increased blood cholesterol level	10 (10.5)
Increase in hepatic enzymes	9 (9.5)
Metabolism and nutrition disorders	7 (7.4)
Infections and infestations	5 (5.3)
Upper respiratory tract infections	3 (3.2)
Influenza	1 (1.1)
Candidiasis	1 (1.1)
Patients with treatment-related SAEs	7 (7.4)
Metabolism and nutrition disorders	3 (3.2)
Hypercholesterolemia	3 (3.2)
Hypertriglyceridemia	1 (1.1)
Investigations	2 (2.1)
Prominent increase in ALT	1 (1.1)
Increased liver enzymes^a^	1 (1.1)
Increased blood cholesterol and LDL levels^a^	1 (1.1)
Infections and infestations	1 (1.1)
Mandibular infection with extension to cervicofacial soft tissue	1 (1.1)
Musculoskeletal and connective tissue disorders	1 (1.1)
Relapse of RA	1 (1.1)

AE, adverse event; ALT, alanine transaminase; LDL, low-density lipoprotein; RA, rheumatoid arthritis.

Data are presented as *n* (%), where *n* = number of patients reporting an event. Multiple occurrences of the same adverse event were counted only once.

One patient with hypercholesterolemia also had hypertriglyceridemia.

^a^Both SAEs occurred in the same patient.

**Table 3:** Laboratory parameters.

Hematology parameter	Mean (SD) changes from baseline to week 24	*P* ^∗^
Hemoglobin, g/dL	0.77 (1.16)	0.001
Lymphocytes, %	9.72 (16.44)	0.001
Neutrophil count, ×10^3^/L	−1.60 (2.48)	0.0001
Platelet count, ×10^3^/*μ*L	−99.7 (73.0)	<0.0001
White blood cell count, ×10^3^/*μ*L	−1.58 (2.46)	0.0001
Albumin, g/L	−1.56 (6.91)	0.005
Alkaline phosphatase, IU/L	−20.7 (28.8)	0.009
Indirect bilirubin, *μ*mol/L	2.43 (3.88)	<0.0001
Total bilirubin, *μ*mol/L	3.42 (5.6)	<0.0001
Total protein, g/L	−3.04 (5.51)	0.0001

SD, standard deviation.

^∗^ *P* ≤ 0.0001 was considered statistically significant based on the Friedman test (analysis of variance).
